# High-risk human papillomavirus in the oral cavity of women with cervical cancer, and their children

**DOI:** 10.1186/1743-422X-7-131

**Published:** 2010-06-16

**Authors:** Rajan Saini, Tan P Khim, Sarah A Rahman, Mazian Ismail, Thean H Tang

**Affiliations:** 1 School of Dental Sciences, Universiti Sains Malaysia, Kubang Kerian, 16150, Kelantan, Malaysia; 2 Infectious Disease Cluster, Advanced Medical and Dental Institute, Universiti Sains Malaysia, Bertam, 13200, Penang, Malaysia

## Abstract

**Background:**

Association of High-risk Human Papillomavirus (HR-HPV) with oral cancer has been established recently. Detecting these viruses in oral cavity is important to prevent oral lesions related to them. The purpose of this study was to evaluate the prevalence of HR-HPV in the oral cavity of women with cervical cancer, and their children. A total of 70 women, previously diagnosed with cervical cancer, and 46 children of these women, born by vaginal delivery only, were selected for this study. Buccal swabs were collected from their oral cavity and HPV detection was carried out using Hybrid Capture 2 high-risk HPV (HC2 HR-HPV) detection system.

**Results:**

Out of 70 women with cervical cancer, four (5.71%) were found to be positive for HR-HPV in their oral cavity. No association of HR-HPV was found with sociodemographic profile, marital status, reproductive history, tobacco and alcohol usage, contraceptive pills usage, and presence of oral lesions (p>0.05). Among children, HR-HPV in the oral cavity was detected in only 1 of the 46 subjects examined (2.17%). Clinically healthy oral mucosa, without any oral lesions, was observed in all the HR-HPV positive subjects.

**Conclusion:**

The result of this study showed that there is low, if any, risk of HR-HPV infection in the oral cavity of women with cervical cancer. Further, our study suggests that there is very low risk for children of women with cervical cancer, to acquire and sustain HR-HPV in their oral cavity until childhood or adolescence.

## Background

Human Papillomavirus (HPV) is an epitheliotropic, double stranded, circular DNA virus from Papovavirus family [1], which is found to infect cells in the basal layer of squamous epithelium [2]. Thus, infection caused by HPV is found in various body sites, such as anogenital tract, skin, conjunctiva, larynx, tracheobronchial mucosa, esophagus and oral cavity [3]. Over the years, more than 130 types of HPV have been identified according to the nucleotide sequence alignment of its open reading frames [4]. HPV is also classified as low-risk and high-risk type, depending on its potential to cause malignant lesions such as cervical carcinomas [5]. In up to 92% of cervical malignancies, certain types of high-risk (HR) HPVs have been identified [6]. HR-HPV oncoproteins (E6 & E7) act by disrupting the function of tumor suppressor genes (pRb & p53), leading to excessive cell growth [1].

A recent meta-analysis has established HPV as an independent risk factor for oral carcinomas as well [7]. Our recent study done in 105 oral squamous cell carcinomas (OSCC) affecting Malaysian population also found HPV to be significantly associated with OSCCs (P < 0.001, OR = 4.3) [8]. As seen in anogenital cancers, HR-HPV 16 is the most common HPV type found in oral carcinomas. Other oncogenic types seen in oral carcinomas include HPV 18, 31 and 33 [9]. Thus, it is imperative to detect HR-HPV in the oral cavity, as otherwise, these might cause benign or malignant HPV related oral lesions like papillomas and oral squmaous cell carcinomas in future. Several studies have been carried out to obtain the relationship of concurrent infection by HPV in cervical and oral sites [10,11].

Different methods have been used to detect HPV in concurrent infections giving varied results. PCR is used in many studies and it is known to be very sensitive in detection of HPV [12]. The *Digene *HC2^® ^assay is United States Food and Drug Administration (USFDA) approved commercially available kit. HC2^® ^assay is a nonradioactive, immuno-chemiluminescence method that is based on the hybridization of genotype-specific RNA probes to HPV genomic sequence. Compared with PCR, HC2^® ^has excellent clinical sensitivity, almost up to 100%, as it shows only positive result when risk of disease progression exists. Studies have shown that HC2^® ^is comparable to PCR and thus, it can be used as an adjunct or stand-alone test in HPV detection [13].

While the HPV related genital lesions are more frequently seen in adults, lesions like skin warts, oral and laryngeal papillomas are more frequently seen in children [14]. Further, the mode of viral transmission in children remains contentious. Several potential modes of transmission have been proposed for these pediatric HPV infections, which include non-sexual routes, like perinatal vertical transmission, auto- and hetero-inoculation, indirect transmission via fomites, and sexual ways, like sexual abuse [15,16]. Hajek suspected vertical transmission of juvenile onset recurrent respiratory papillomatosis (RRP), a relatively rare disease caused by HPV, from a mother to her child at birth, as early as 1956 [17]. Since then, several studies have been published giving results which vary from as low as 0% to as high as 80% [18-20]. An argument against these earlier studies was that as the samples were taken at the time of, or soon after delivery, the HPV that was being detected in such samples may actually reflect a surface contamination of the infant with HPV infected maternal cells, rather than infection itself [21].

Therefore, the purpose of this study was to detect the prevalence of HR-HPV in the oral cavity of women with cervical cancer, and to evaluate the risk factors which contribute to its occurrence. Also, this study aimed to detect the existence of HR-HPV in children of the women with cervical cancer, for evaluation of vertical transmission of this virus, and to assess any associated risk factors.

## Results

### Demographic profile of women with cervical cancer, and their children

In total, 70 women diagnosed with cervical cancer and 46 children of these women, were recruited for this study. The demographic profile of the women and their children is given in Table 1 and Table 2 respectively. More than two-third of the women examined (68.5%), were above 50 years of age, with the mean age of 55.21 years. Most of the subjects were Malay (82.9%) and married (84.3%). Most of the women did not have any tobacco or alcohol habits. For children, 82.6% were from 11 to 18 years of age, with the mean age of 14.78 years. There were more female children (54.3%) than males (45.7%). Most of the children did not have any tobacco or alcohol habits.

**Table 1:** Demographic profile of women with cervical cancer

Variables	n (n = 70)	%
**Age range**		
30-39	4	5.7
40-49	18	25.7
50-59	22	31.4
60 & above	26	37.1
Mean ± SD	55.21 ± 9.57	
**Race**		
Malay	58	82.9
Chinese	11	15.7
Indians	1	1.4
**Marital status**		
Married	59	84.3
Single	1	1.4
Divorce	2	2.9
Remarried	8	11.4
**Age when married**		
<18 years old	33	47.1
> 18 years old	37	52.9
**Number of children**		
< 5	26	37.1
>5	44	62.9
**Habits**		
Tobacco	3	4.3
Alcohol	4	5.7
**Pregnancy and contraception**		
Pregnant	2	2.9
OCP usage	24	34.3

**Table 2:** Demographic profile of children of women with cervical cancer

Variables	(n = 46)	%
**Age range**		
1-5	2	4.3
6-10	6	13.0
11-15	16	34.8
16-18	22	47.8
Mean ± SD	14.78 ± 4.69	
**Race**		
Malay	44	95.7
Others	2	4.3
**Gender**		
Male	21	45.7
Female	25	54.3
**Habits**		
Tobacco	3	6.5
Alcohol	1	2.2

### HR-HPV in the oral cavity of women with cervical cancer, and their children

Four samples (5.71%) from the oral cavity of women with cervical cancer showed positivity for HR-HPV using HC2^® ^detection system, with RLU/CO value being >1. Among the children, only 1 (2.17%) sample was found to be positive for HR-HPV in the oral cavity. All the positive subjects showed clinically healthy oral mucosa without any lesions. Association of HPV in cervical cancer subjects was evaluated with age range, ethnicity, marital status, age when got married, number of children, pregnancy status, contraceptive pills usage, tobacco and alcohol habits, and any oral lesions. Chi square/Fisher's Exact test showed no significant association between HR-HPV infection with any of these variables (p > 0.05) (Table 3). As the percentage of HPV positivity was very low in children (1/46, 2.17%), no statistical analysis was performed.

**Table 3:** Association of HR-HPV in the oral cavity of women with cervical cancer, with variables

Variable	n	HPV positive n (%)	X^2^	*p *value^a^
Age range				
30-39	4	0 (0)	4.06	-
40-49	18	0 (0)		
50-59	22	3 (13.6)		
60 & above	26	1 (3.8)		
Ethnicity				
Malay	58	2 (3.4)	3.22	0.133
Others	12	2 (16.7)		
Marital Status				
Married	59	4 (6.8)	0.79	-
Single	1	0 (0)		
Divorced	2	0 (0)		
Remarried	8	0 (0)		
Age when married				
<18 years old	33	1 (3.0)	0.84	0.352
> 18 years old	37	3 (8.1)		
No of children				
< 5	26	2 (7.7)	0.30	0.476
>5	44	2 (4.5)		
Pregnant				
Yes	2	0 (0)	0.13	0.888
No	68	4 (5.9)		
OCP usage				
Yes	24	0 (0)	2.21	0.178
No	46	4 (8.7)		
Tobacco usage				
Yes	3	0 (0)	0.19	0.836
No	67	4 (6.0)		
Alcohol consumption				
Yes	4	0 (0)	0.26	0.786
No	66	4 (6.1)		
Oral lesions				
Yes	6	0 (0)	0.398	0.693
No	64	4 (6.3)		

^a^Fisher's- exact test

## Discussion

Various studies have been conducted to study the role of HPV in oral lesions and malignancies. However, the association of HPV between cervical and oral cavity remains unclear. Further, recent studies on mother-to-child transmission by perinatal infection with HPV have been inconclusive [15]. Considering the fact that almost all the cervical cancers are caused by HPV [22], this study was conducted to evaluate the prevalence of HR-HPV in the oral cavity of women with cervical cancer, and their children. To our knowledge, this is the first study that has been carried out simultaneously in women with cervical cancers, and their children, to detect the presence of HR-HPV in their oral cavity.

Our results showed the prevalence of HR-HPV in oral cavity of women with cervical cancer to be quite low, with only 4 out of 70 subjects (5.71%) testing positive for HR-HPV. The results found in this study were in concordance with the study done by Kellokoski *et.al *[23], which examined the cytological scrapings of oral mucosa in 309 women with genital HPV infections, by using dot blot hybridization and found oral HPV infection in only 3.8% women. Of these, only 2 had clinical lesions suggestive of HPV infection. In our study, all the HPV positive subjects had clinically normal oral mucosa. Another study, that was done to determine the HPV prevalence and concurrent infection in the cervix and oral cavity of 577 pregnant women, found 29% positivity in the cervix and 2.4% positivity in the oral cavity. No association was found between HPV positivity and its types detected in the cervix and oral cavity of these women, suggesting that self-inoculation was rare [24].

While recent studies have shown the presence of HR-HPV in a faction of oral pre-malignant and malignant lesions, this may suggest a role of HPV in only a portion of oral malignancies, contrary to cervical region, where its association is noted in almost all the cervical malignancies [25]. This difference in HPV invasiveness could be due to various factors. Firstly, oral cavity is in direct contact with carcinogens present in tobacco and alcohol, making them the primary cause of oral carcinogenesis. This is not the case with cervical region, where there is no direct contact with these carcinogens. Secondly, the low prevalence of oral HPV infection might be due to the body's immune response, like immunoglobulin IgA and proteolytic enzymes in the saliva that protect the oral mucosa from viral infections [26]. Thirdly, antibodies produced by the body in response to initial infection, in this case cervical infection, might as well protect the body against further infections by the same virus on other sites. Fourthly, although the oral mucosal epithelium resembles the epithelium of the genital tract [27], antimicrobial action of saliva, along with its cleansing and lubricating properties, may reduce the possibility of virus entry into the oral epithelial cells by reducing the contact period of the virus with the oral mucosa [28]. Finally, considering the HPV detection method, although cytological scraping has many advantages, like being painless and non-invasive method, thus having better patient compliance compared to invasive procedures like biopsy, the disadvantage of this technique is that, in scrapings, basal and parabasal cells cannot be collected, which could lead to false negative results. Failure to collect the infected cells from basal layers might have also contributed to the low prevalence found in our study.

In contrast to our findings, a higher percentage (15.4% and 29.4%) of concurrent HPV infection between clinically normal oral mucosa and genital region, by using Southern blot and PCR procedures respectively, was obtained by Kellokoski *et.al *[29]. In another study, Badaracco *et.al *studied concurrent HPV infection in oral & genital mucosa by using PCR based assay. Sixteen subjects positive for HPV (31.25%) showed simultaneous genital & oral cavity infections, while HPV type-specific concordance was detected in only 3 patients [10]. These differences in prevalence could be due to sensitivity of the assays used, and differences in sample size. Most of the previous studies were conducted using PCR based assays for HPV detection. PCR is highly sensitive with detection limits between 10-400 copies of HPV DNA resulting in detection of clinically insignificant viral levels which can be cleared by our own immune system. HC2^®^, on the other hand, detects clinically relevant viral levels, that is 5000 copies and above [30]. At these viral levels, the probability of developing HR-HPV disease is high. Thus, prevalence obtained in studies that used PCR as HPV DNA detection assay will be higher as compared to HC2^®^. Moreover, HC2^® ^detects only 13 types of HR-HPVs, while PCR based assays detect all types of HPVs.

Our study did not find any association of HPV in cervical cancer subjects with age range, ethnicity, marital status, number of children, tobacco or alcohol usage, contraceptive pills usage, or any oral lesions. This could be due to small percentage of positivity seen in our results and smaller sample size. Studies have shown that smoking has potential to alter oral epithelium, thus it has an influence on HPV expression in oral cavity [31]. However, as most of the subjects in this study (94.29%) were non-smokers and non-alcoholics, any association with oral habits could not be established.

This study did not assess the presence of HPV in the genital regions of women with cervical cancers at the time of examining their oral cavity for HR-HPV. It is because this study was more focused on detecting HR-HPV in the oral cavity, which might cause benign or malignant HPV related oral lesions in their future, rather than detecting concurrent infections in genital and oral regions, as reported in previously mentioned studies.

This study also aimed to detect the presence of HPV in oral cavity of the children of these women. Only 1 of the 46 children analysed was found to be positive for oral HR-HPV (2.17%). Further questioning from this HR-HPV positive subject revealed that he was sexually active. Thus, the positivity could be, in part, due to the sexual activities rather than transmission from the mother. Similar to our results, Koch *et al *tested the presence of HPV in the anal region and the oral cavity of Danish children, aged 0 to 17 years, by PCR targeting the L1 region of the HPV genome. Only four of the 249 anal samples and one of 392 oral samples were found to be HPV positive. The authors concluded that ano-genital types of HPV are not transmitted by non-sexual routes, and that HPV infection mainly occurs later in life [32].

Children of older age groups were chosen for analysis in our study. This is because the infants were shown to have a higher percentage of oral HPV, which could be due to the result of surface contamination that might occur in these infants during childbirth rather than HPV infection per se [33]. This finding was reconfirmed by a recent study in which all the HPV-DNA positive newborns (22.4%) at birth and at the end first month of life (6.1%) became HPV-DNA negative by the age of 6 months [34]. Similarly, in 1986, Roman and Fife analysed the foreskins of 70 male infants undergoing routine circumcision for HPV type 6, 11, 16, and 18, by using dot blot hybridization and found HPV in 4% of the cases. The result suggested that neonates exhibit a relatively high incidence of exposure to HPV during or before birth. However, no correlation could be identified between mothers with abnormal pap smears and the HPV-positive foreskins [35]. Another recent study analysed 49 HPV DNA-positive pregnant women at the time of delivery and found 24.5% placentas had a positive result for HPV DNA. Eleven newborn were HPV DNA positive in samples from the nasopharyngeal or buccal and body or cord blood. Out of these, 5 cases (10.2%) had HPV type-specific agreement between genital/placenta/newborn samples suggesting transplacental transmission [36]. Other studies have also supported the concept that maternal genital-tract HPV infection could cause other diseases like respiratory [37] and laryngeal papillomas [38] in children. Oral HPV prevalence in children has also been reported by few researchers [39,40]. Studies on newborn babies have detected a higher prevalence of 37% to 73% HPV DNA in the nasopharyngeal aspirates or buccal swabs [41-43].

Our study was cross-sectional as compared to several other studies which were longitudinal, starting from the HPV detection since the mother became pregnant, until the delivery or a few months after birth [20,21,44,45]. Based on our results, which showed very low HR-HPV positivity, we presume that even though there is a probability that HPV can spread to the oral cavities of children via vertical transmission, it will not persist until their adulthood. Most likely, the HPV DNA that detected right after their delivery was due to contamination, as suggested in other studies.

## Conclusion

This study shows that there is low, if any, risk of HR-HPV infection in oral cavity of women with cervical cancer. There were no relevant risk factors that contributed to the development of this infection. Further, our study suggests that there is very low risk for children of women with cervical cancer, to acquire and sustain HR-HPV in their oral cavity until childhood or adolescence. More studies with bigger sample size are recommended to determine the relationship of HPV infection in these two areas. For more accurate evaluation of vertical transmission, long term follow-up studies should be conducted.

## Methods

### Study Design and data collection

This was a cross-sectional study that involved 70 women who were diagnosed to have cervical cancer, undergoing active treatment and routine follow up in Obstetrics and Gynaecology department or Oncology department of Hospital Universiti Sains Malaysia. A total of 46 children of these women, born only with vaginal delivery, were also examined. Only those children, who were born after the women were suspected or diagnosed of cervical cancer, were recruited in this study. Ethical approval was obtained from the Human Research Ethics Committee of Universiti Sains Malaysia. After obtaining the consent and explaining the procedure to each participant, questions about sociodemographics, marital status, reproductive history, tobacco or alcohol usage, contraceptive pills usage, oral and medical health status were asked using questionnaire provided to women at the time of enrollment. The questionnaire given to children was to inquire about their sociodemographic profile and habits. The children, who were older than 18 years of age, were married, or in any immunocompromised state like diabetes, were excluded from this study. Oral cavity of the subjects was also checked for any oral lesions, including HPV related oral lesions like papillomas, condylomas and focal epithelial hyperplasia, by a single oral medicine specialist to avoid inter-examiner variability.

### Sample collection and testing

The buccal swabs were collected using DNAPap Cervical Sampler™, from right and left buccal mucosa by moving the brush in circular motions, and then kept in the transport medium. All the sample tubes were stored immediately in -20°C until testing for HPV. All collected specimens were tested using Hybrid Capture 2^® ^(HC2) high-risk (HR) HPV DNA detection system located in the Department of Pathology, Universiti Sains Malaysia, and the procedure followed was according to the instruction of manufacturer. *Digene *HC2^® ^detects HR- HPV (Qiagen, U.S.A.) by using RNA probe cocktails to detect 13 HPV-HR which are HPV type-16, 18, 31, 33, 35, 39, 45, 51, 52, 56, 58, 59 and 68. HC2^® ^technology is a nucleic acid hybridization assay for detection of HPV with signal amplification using microplate chemiluminescent detection. Light emitted is measured in terms of relative light units (RLUs) using Luminometer. RLU value is converted into ratio by the Cutoff value (5000 copies/ml). Any specimen with RLU/CO ≥1 was considered positive.

### Statistical Analysis

The prevalence of HPV in the oral cavity of cervical cancer subjects was analysed using estimation method. The association of HPV infection with risk factors (sociodemographic profile, marital status, reproductive history, tobacco and/or alcohol habits, contraceptive pills usage and any oral lesions) was analyzed using Chi square test/Fisher's exact test with software SPSS version 16.0.

## Competing interests

The authors declare that they have no competing interests.

## Authors' contributions

RS designed the research project and drafted the manuscript. TPK collected and processed the samples from the women with cervical cancer. SBR collected and processed the samples from the children of the women with cervical cancer. RS and TTH guided the bench work of the procedure. MI did the bench work for detecting HR-HPV in samples. RS and TTH critically reviewed the final manuscript. All authors read and approved the final manuscript.
